# Residual Monomer Content Affects the Interpretation of Plastic Degradation

**DOI:** 10.1038/s41598-019-38685-6

**Published:** 2019-02-14

**Authors:** Franziska Klaeger, Alexander S. Tagg, Stefan Otto, Matthias Bienmüller, Ingo Sartorius, Matthias Labrenz

**Affiliations:** 1 Leibniz Institute for Baltic Sea Research Warnemünde (IOW), Rostock, 18119 Germany; 2 Lanxess Deutschland GmbH, Dormagen, 41538 Germany; 3 PlasticsEurope Deutschland e.V., Frankfurt am Main, 60329 Germany

## Abstract

Plastic degradation rates in the marine environment are essential to understand. This study demonstrates that in plastic-microbial interaction experiments, residual monomeric and oligomeric content of PA6 significantly influences the development of dissolved organic carbon. While it is well recognized that additives in plastics should be considered during the inception of plastic-exposure experiments, residual monomers have yet to be prominently considered in the same light. As such, in degradation studies where residual contents of monomers and/or oligomers are not considered, degradation of synthetic polymers could be significantly overestimated. The substantial conversion of these monomeric and oligomeric leachates also has implications for plastic-biofilm development studies and microplastic-biota-based ingestion experiments.

## Introduction

There are well-established and worldwide concerns about plastic pollution. As such, it is essential to understand the potential for degradation of plastics in the environment, especially the marine environment. Plastic degradation is defined as any physical or chemical change in the polymer resulting from light, heat, moisture, chemical action or biological activity that results in a decrease in the average molecular weight^1^. Typically, plastic degradation results in fragmentation of larger plastic pieces into smaller plastic particles by a combination of abiotic (such as UV irradiation) and biotic factors (extracellular enzymatic action resulting in biodeterioration/biofragmentation). When this process results in particles small enough to be assimilated by microorganisms, the final process of plastic degradation involves intracellular conversion into simple molecules such as CO_2_^2,3^. Degradation tests for polymers in the marine environment are very specific and standardization procedures are not yet fully developed^4^. Another factor complicating research into plastic degradation is the complexity of plastic materials with regard to possible structures and compositions, making the testing of plastic degradability a highly interdisciplinary process^5^. Abiotic degradation and biodeterioration are usually investigated using physical tests. Biofragmentation is determined by the identification of fragments with lower molecular weight using chromatographic methods. Assimilation is often measured by the metabolite production or the increase of biomass. Mineralization is typically measured by ascertaining changes in either dissolved organic carbon, biological oxygen demand or CO_2_ evolution (see Lucas *et al*.^3^).

Specifically designed biodegradable plastics notwithstanding, standard and engineering polymers (typically long-chain molecules from polymerisation, polycondensation or polyaddition) are generally considered to be resistant to biodegradation. The polyamides (PA) are one such important synthetic polymer belonging to the group of engineering plastics. PAs are considered to be biodegradable-resistant polymers^6,7^, although some studies have demonstrated the biodegradation potential of some polyamides, particularly by fungi^8,9^. However, it may be that increases in carbon evolution, oxygen demand or biomass may be the result of microbial metabolism of residual monomers or oligomers, rather than the polymer chain. For example, PA6 (commonly referred to as nylon-6) is produced by ring-cleavage polymerization. During this process, some molecules fail to polymerize and remain as oligomers and monomers within the structure of the polymer. Although these components are by-products rejected by the producing factory^10^, a small amount typically stays within the polymer. Depending on the further use of the plastic, manufactured polymers contain different amounts of residual monomers and oligomers (rM). The raw material of the PA6 tested within this study is the monomer ε-caprolactam, which is known to be biodegradable by microorganisms found in the wastewater produced by PA6-production facilities^11–13^. It is possible that rM in the tested PA6 could have been much more easily metabolised than polymeric content by microorganisms and therefore could lead to false-positive interpretations of polymer biodegradation. This study firstly investigated whether an indication of PA6 degradation could be achieved following incubation in a seawater analogue. In order to understand the effect that rM content could have on the interpretation of plastic degradation, exposure tests investigating differing monomer contents were designed where dissolved inorganic carbon (DIC = [CO_2_] + [HCO_3−_] + [CO_3_^2−^]) was selected as the indicator by which biological degradation is chemically measured.

## Results

### Investigating plastic degradation in seawater

PA6 (poly(ε-caprolactam) was incubated in microbial-rich artificial brackish water (ABW; see Methods section for details). Any increase in DIC after a certain incubation time could be, in theory, assigned to mineralization of carbon from the plastic by microorganisms.

To ensure that the measured carbon came from the plastic, the ^14^C content was determined in the DIC of the ABW following incubation. Synthetic polymers originate from petrochemicals in which, due to the radioactive decay, ^14^C is absent. There are three principal isotopes of carbon which occur naturally in the following amounts: ^12^C at 98.89%, ^13^C at 1.11% and ^14^C at 10^−10^%. A lower ratio of ^14^C compared to ^12^C and ^13^C in the PA6 incubation media can be seen as proof for mineralization of the plastic substrate.

**Figure 1:**
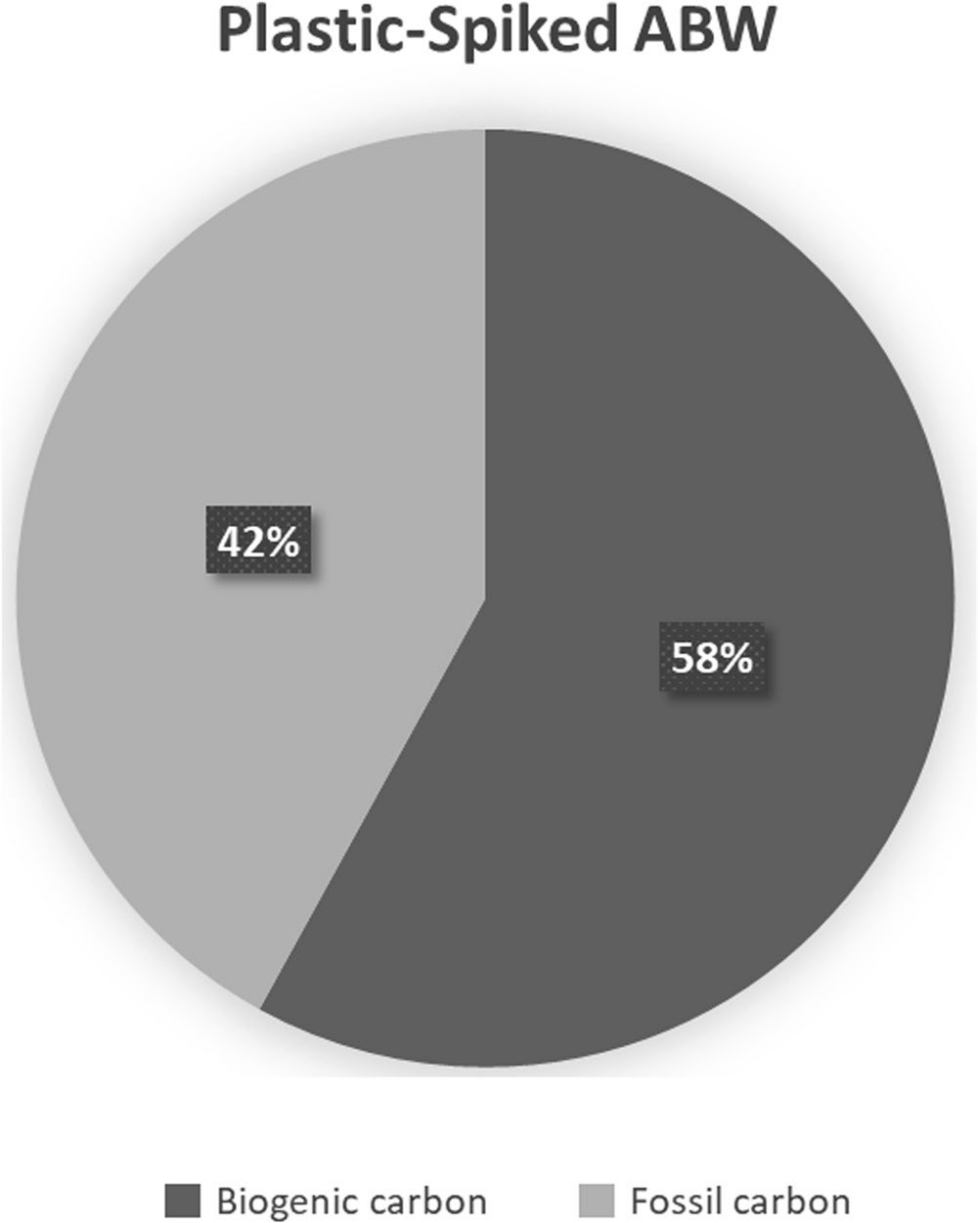
Carbon isotope ratio breakdown detected by AMS in incubation media inoculated with PA6 (~1**%** rM). At least 42**%** of the carbon detected in the incubation media originated from the PA6 spiked-particles. In comparison, a natural setup typically exhibits 102**%** biogenic carbon.

A clear difference was observed in the carbon isotope ratio of PA6-incubated ABW compared to today’s biogenic carbon standard reference of ~102 percent Modern Carbon (pMC) (Fig. 1). PA6-incubated ABW exhibited only 58% (59.0 +/− 0.2 SE pMC) of carbon which originated from biogenic sources. 42% of the carbon can therefore be considered of fossil (i.e. plastic) origin. Thus, results suggest carbon originating from PA6 plastic had transferred into the ABW medium.

### Residual monomer content on DIC development

PA6 particles of 3 different rM concentrations were investigated (~1%, ~0.1%, ~0.05%). In addition to monomer content, particle size and exposure time (in ABW) were also examined (see Methods section for details). The development of DIC was compared to both starting values and control values (ABW incubation without PA6).

Particle size was found not to be significant (K-W test; n = 3, g = 5, df = 4; *p* = 0.06). However, given to proximity to the significance threshold (*p* = 0.05), *post hoc* analyses were undertaken (Dunn’s test). These results showed that, between all size groups, one significant difference was apparent between size classes A and B (see methods section). On close examination, 2 DIC measurements appeared to be unusually high in the size group B. Since DIC measurements for all other groups, including both smaller and larger particle-groups, were much lower (and statistically similar), these values were considered erroneous. When group B was discounted, particle size clearly had no significant effect on DIC measurement (K-W test, n = 3, g = 4, df = 3; *p* = 0.3254). Different incubation time also proved to have no significant impact on the DIC development (1-way ANOVA; n = 6, g = 3, df = 2; F = 1.397, *p* = 0.278). As such, both size and exposure time were considered statistically unimportant variables for downstream analyses.

**Figure 2:**
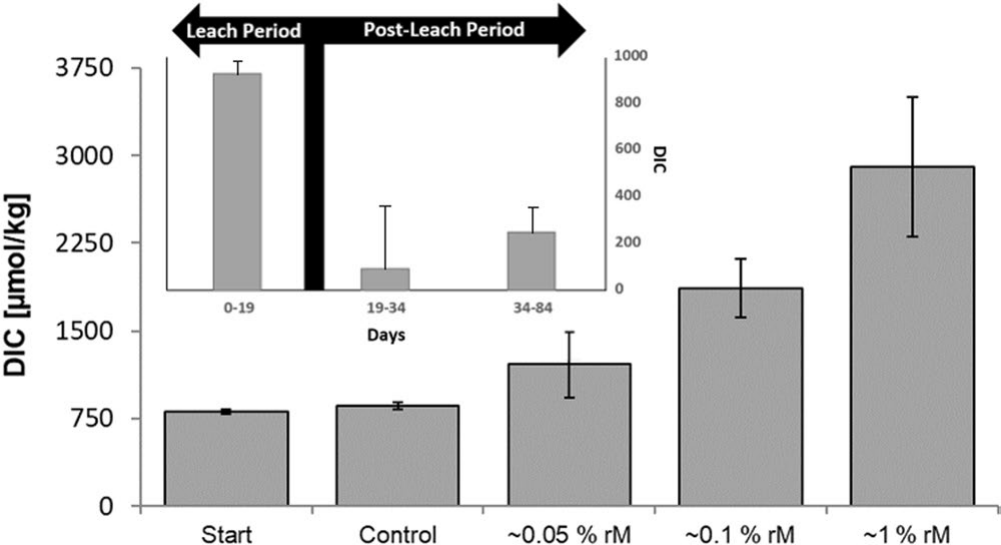
Increases in dissolved inorganic carbon (DIC) can be seen following microbial exposure to PA6 of varying residual monomer (rM) content. Results can be seen compared to the starting DIC of the initial exposure setup as well as the control which represents the exposure setup without PA6. Top-Left Inset: DIC development of PA pellets with 0.1% rM content during the peak leaching period (first 19 days; similar to that described by Romera-Castillo *et al*.)^17^ compared to DIC development following this period.

PA6 rM content had a highly significant effect on DIC development (Fig. 2; 1-way ANOVA; n = 9, g = 3, df = 2; F = 33.59, *p* = 1.13 × 10^−7^). *Post hoc* analyses (Tukey HSD) showed that all rM content groups were significantly different. Moreover, the strength of the statistical difference followed the pattern of monomer content, with the weakest significance (*p* = 0.03) between the two most similar monomer content groups (~0.1% rM and ~0.05% rM), while the difference between groups ~0.1% rM and ~1% rM being highly significant (*p* = 4.96 × 10^−5^) and the difference between ~1% rM and ~0.05% rM (the largest difference in rM content tested) giving the strongest significant interval (*p* = 0.01 × 10^−5^). An increase of DIC was observed in all PA6 samples compared to starting and control values (see Fig. 2). As such, this result is consistent with the findings of the prior ^14^C analysis. Given the identical experimental setup, this further strengthens the finding that carbon originating from the PA6 particles was biologically mineralized, and that, given the results of the second experiment, a prominent component of this appears to be attributed to the mineralization of monomeric and oligomeric content.

## Discussion

Using isotopic ratios as an indicator to test for degradation is known in combination with radiolabelling the test substance. Radiolabelling is often used when slowly degradable materials are to be investigated^14,15^. The radiolabelling of the polymer is used in several standardized analytical methods, such as by the Organization for Economic Co-operation and Development (OECD) in the test No. 304^16^. However, labelling the polymer substrate is expensive and working with radioactive substances is experimentally challenging. By investigating the ^14^C in the incubation media, as demonstrated in the preliminary experiment, the expensive and challenging labelling can be avoided whilst degradation of polymers can still be experimentally demonstrated. However, when using this procedure, it is essential to know the rM content of tested virgin plastics. This is because rM content was shown to have a strong effect on the amount of DIC development.

Unfortunately, it was not possible to distinguish whether the observed DIC increase originated from abiotic or biotic effects. However, it is unlikely to be considerably abiotic, as no abiotic factors, such as high temperatures or UV radiation^1^, were applied during the incubation period. Moreover, if abiotic factors were somehow significant, results would have nevertheless been the same for all treatments and therefore does not affect the interpretation of the results. Furthermore, this research is intended to be an example of typical experimental setups which are designed to imitate natural environmental processes. Therefore, since abiotic and biotic effects are both important factors when considering the natural degradation of plastics in the environment, the main finding of this experiment, that higher rM content favours higher environmental degradation interpretation, is a highly relevant one.

Interestingly, within this experiment, neither exposure time nor particle size significantly influenced the development of DIC in the exposure mediums. A recent study by Romera-castillo *et al*.^17^ may explain why these variables had no significant bearing on results. The authors investigated the effect of leached dissolved organic carbon (DOC) from plastics on the surrounding microbial community and found that the maximum rate of DOC leaching occurs immediately, with 60% of this available for microbial utilization after 5 d. They also observed that DOC leaching rates had plateaued after around 10–16 d. Since in our study, the first DIC measurement was made after 19 d exposure, the greatest leaching and consequent microbial response likely already occurred, with consequent time points (34 and 84 d) well past the probable DIC-leaching plateau-point. Based on the findings of Romera-Castillo *et al*.^17^, exposure time is likely to have an important effect on DIC leaching when shorter time-frames are considered. This effect also likely explains why size is equally shown to be insignificant within the present study.

While shorter time-framed investigations into the effect of rM content on DIC development would be interesting to reveal the leaching patterns of monomeric and oligomeric content, the results of this study concerning the strong effect that rM content has on DIC development cannot be understated. Any study that investigated plastic degradation patterns of polymers without considering rM content may well have overestimated early polymer degradation rates. The measurement of development in DIC or DOC, biological oxygen demand, metabolite production or biomass increase could all be a result of early monomer leaching and subsequent microbial utilization. For example, an early (1988) and highly cited paper investigated the degradation stages of polyethylene (PE) by measuring the development of ^14^CO_2_ in soil originating from ^14^C-labelled plastic matrix following incubation^18^. While this was based on a much longer time frame (10 years), the authors nevertheless described an early, rapid degradation stage before rates settled to a consistent, low, ^14^C conversion rate. It is possible that a reasonable amount of early ^14^C conversion was the result of monomeric and oligomeric conversion, the level of which could be affected by essential factors like polymer type (PE-HD, PE-LD or PE-LLD, etc.) or the additive stabilisation of the polymer. Therefore, this early degradation stage may not actually represent environmental polymer degradation.

PA was used as an exemplary polymer within this study. However, as mentioned, other attributes of the plastic may influence the described rM affect. Certain polymers may be expected to demonstrate similar results to PA. The monomers styrene, vinylchloride (VC) and bisphenol-A (BPA) have all been shown to leach from the plastics polystyrene (PS), polyvinylchloride (PVC) and polycarbonate (PC) respectively^19–22^. Indeed, recent research has shown that initial leaching and microbial conversion of the monomer bisphenol-A (BPA) from virgin polycarbonate also occurs at highest rates within the first 20 days^23^, a similar leaching period observed in this study and also described by Romera-Castillo *et al*.^17^. In addition to polymer type, other features, such as the average molecular weight, toughness or amorphous/crystalline ratio could have an influence on the rate of rM leaching (and thus the development of DIC or some other degradation measure).

It is essential that any environmental degradation study considers the implications of early rM leaching to prevent false-positive interpretation of polymer degradation. In addition, care should be taken on specifics of the investigated polymer type, such as the exact material type (including physical constituency, stabilisation, etc.) where the use of gel permeation chromatography (GPC) may be of particular value for future research into such properties^24^. Additionally, care should be taken when preparing any respective environmental media, where a thorough and careful preparation, such as the ABW used within this study, is preferred.

The impact of rM content in virgin-polymer (recently manufactured from either newly synthesised stock or from secondary raw materials, i.e. plastic recyclates) exposure studies not only has implications for interpreting plastic degradation rates. Microbial-associated exposure studies could be greatly affected by the monomeric and oligomeric content of polymers used, particularly those interested in the early microbial colonization of recently-manufactured plastics and microplastics^25,26^. Given that plastic leachates have already been shown to have a considerable influence on surrounding microbiota^17^ and that leaching occurs immediately, the effect of rM content on microbial colonisation and early biofilm development must be considered in future experimental designs. Additionally, while the toxicity of polymer ingredients are well defined according to EU substance legislation, especially REACH and CLP Regulation, there are still some concerns over certain monomers^27–31^. Therefore, the effects of available rM during plastic-ingestion experiments must also be considered.

As such, the results of this study show that, based on PA as an exemplary polymer, rM content is a highly important factor, and prior interpretations of plastic degradation where this parameter is not correctly controlled for, may therefore, overestimate polymer degradation. Additionally, both microbial biofilm development and microplastic effects on biota may also be affected by the rM content of the tested polymer. For this reason, rM must be considered in the experimental setup of future research in these areas.

## Methods

### Investigating plastic degradation in seawater

Additive-free PA6 (poly(ε-caprolactam) powder (LANXESS, *Dormagen*, *Germany*) was selected for testing. A size of <125 µm was achieved by cryo-grinding with liquid nitrogen in a pin mill and sieving through a vibrating sieve. Prior to the experimental start, the PA6 was exposed to UV light for 16.5 hours to better align virgin PA6 samples with environmental microplastics. PA6 was then incubated in ABW; modified after Bruns *et al*.^32^ (see supplementary information) for 47 days. The ABW was produced, where possible, avoiding carbon sources. Prior to incubation, Baltic Sea microorganisms were filtered onto a 0.2 µm filter with two pre-filtration steps through an 100 µm stainless steel sieve and a 10 µm filter from 2x the volume of Baltic Sea water compared to the final volume of ABW produced. All solutions used for the ABW were autoclaved or sterile filtered and stored at 15 °C to the point of use. The ABW was aerated over night with a membrane pump (WISA 200 l/h with 0.1 bar). Per reagent bottle, 7 g of prepared PA6 as described above, was added to 130 ml of ABW. A control sample of microbial-rich ABW without PA6 was included. Samples were incubated at 23 °C in darkness and kept mixed to keep the plastics in suspension. PA6-incubated ABW samples (n = 3) were sent for Biogenic Carbon Content Analysis using the American Society for Testing and Materials (ASTM) D6866-12^33^ standard method (Beta Analytic, *Miami Florida*, *USA*, ISO/IEC 17025:2005 accredited). The 3 replicates were combined in an argon-filled glove box to avoid exposure to air before measuring ^14^C content in the ABW by accelerator mass spectrometry (AMS). With AMS, the ^14^C fraction was measured in relation to the ^12^C and ^13^C occurrences in the DIC and compared to a modern reference standard. As standard reference a NIST (National Institute of Standards and Technology) standard was used with a known radiocarbon content equivalent approximately to the year AD 1950. Today’s samples become corrected with a factor of 0.98 due to nuclear background radiation since 1950. Thus, today’s value for fresh biomass material is ~102 pMC. To facilitate analyses, acidification with phosphoric acid (~53% final concentration) was undertaken.

### Residual monomer content on DIC development

Additive-free PA6 (poly(ε-caprolactam) (LANXESS, *Dormagen*, *Germany*), with differing rM contents (~1%, ~0.1% and ~0.05%; rM concentrations occur at ~10:1 monomers to oligomers in the original ~1% stock) was selected for testing. The reduced rM content of ~0.1% and ~0.05% were achieved by methanol extraction followed by water extraction of stock ~1% rM PA6 using the modified standardized protocol EN ISO 6427^34^ (LANXESS *Dormagen*, *Germany*).

The PA6 and control samples were prepared as in the ^14^C experimental setup (see above). In addition to the standard controls (ABW without PA6), PA6 exposures were performed in triplicate; controls in duplicate (see supplementary material). A comparison of PA6 ~1% rM content with ultrapure water showed only a very minor increase. As such, any immediate increase in DIC introduced at the point of PA6 spiking was considered negligible (see supplementary material). Oxygen content (Hach Lange HQ40d system equipped with a luminescence dissolved oxygen sensor LOD) was measured in parallel reagent bottles to confirm that aerobic conditions prevailed in all samples (see supplementary material). Since only one DIC measurement per bottle is possible, the starting value was measured in 3 separate reagent bottles containing only ABW.

The DIC was measured through coulometric titration with the Single Operator Multiparameter Metabolic Analyser (SOMMA)^35^. In addition to monomer content, both particle size and exposure time were also investigated. To examine the impact of particle size, 5 different size classes (A: >125 µm; B: 125–250 µm; 250–500 µm; D: 500–1500 µm; E: ~4000 µm) of ~1% rM-content PA6 were exposed to inoculated ABW for 34 d. To examine the effect of incubation time, both ~0.1% rM and ~0.05% rM were exposed for 3 time periods (19 d, 34 d and 84 d). The datasets generated and analysed during the current study are available in the supplementary information. Both exposure period data and monomer content were compared using one-way Analysis of Variance analyses (ANOVA) with *post hoc* testing using Tukey’s Honestly Significant Difference (HSD) test. Size data did not conform to the assumptions of an ANOVA, therefore the non-parametric Kruskal-Wallis (K-W) test was performed with *post hoc* testing using Dunn’s test. All analyses were completed using R v. 3.4.2^36^ with the Tukey HSD test performed using the package “agricolae”^37^ and Dunn’s test performed using the package “dunn.test”^38^.

## Supplementary information

Supplementary Information

## References

[1] Andrady AL (2011). Microplastics in the marine environment. Mar. Pollut. Bull..

[2] Andrady AL (1994). Assessment of Environmental Biodegradation of Synthetic Polymers. J. Macromol. Sci. Part C Polym. Rev..

[3] Lucas N (2008). Polymer biodegradation: Mechanisms and estimation techniques. Chemosphere.

[4] Weber, M. *et al*. Assessing Marine Biodegradability of Plastic—Towards an Environmentally Relevant International Standard Test Scheme. In *Proceedings of the International Conference on Microplastic Pollution in the Mediterranean Sea* (eds Cocca, M. *et al*.) 189–193 (Springer International Publishing, 2018).

[5] Müller, R.-J. Biodegradability of Polymers: Regulations and Methods for Testing. In *Biopolymers Online* (ed. Steinbüchel, A.) (Wiley-V. C. H Verlag GmbH & Co. KGaA, 2005).

[6] Ennis DM, Kramer A (1975). A rapid micro technique for testing biodegradability of nylons and related polyamides. J. Food Sci..

[7] Oppermann FB, Pickartz S, Steinbüchel A (1998). Biodegradation of polyamides. Polym. Degrad. Stab..

[8] Deguchi T, Kakezawa M, Nishida T (1997). Nylon biodegradation by lignin-degrading fungi. Appl. Environ. Microbiol..

[9] Klun U, Friedrich J, Kržan A (2003). Polyamide-6 fibre degradation by a lignolytic fungus. Polym. Degrad. Stab..

[10] Negoro S (2000). Biodegradation of nylon oligomers. Appl. Microbiol. Biotechnol..

[11] Nonomura S, Kotani R, Urakabe R, Shima S, Sakai H (1974). A New Metabolite, 6-Oxo-7-azatridecanedioic Acid, a Microbial Product from Cyclic Dimer of ε -Caprolactam. Agric. Biol. Chem..

[12] Kinoshita S, Kageyama S, Iba K, Yamada Y, Okada H (1975). Utilization of a Cyclic Dimer and Linear Oligomers of ε-Aminocaproic Acid by Achrornobacter guttatus KI 72. Agric. Biol. Chem..

[13] Kulkarni RS, Kanekar PP (1998). Bioremediation of ε-Caprolactam from Nylon-6 Waste Water by Use of Pseudomonas aeruginosa MCM B-407. Curr. Microbiol..

[14] Albertsson A-C (1978). Biodegradation of Synthetic Polymers. II. A Limited Microbial Conversion of ^14^C in Polyethylene to ^14^CO_2_ by some Soil Fungi. J. Appl. Polym. Sci..

[15] Tuomela M, Hatakka A, Raiskila S, Vikman M, Itävaara M (2001). Biodegradation of radiolabelled synthetic lignin (^14^C-DHP) and mechanical pulp in a compost environment. Appl. Microbiol. Biotechnol..

[16] Organization for Economic Co-operation and Development (OECD). *Test No. 304A: Inherent Biodegradability in Soil.* (1981).

[17] Romera-Castillo C, Pinto M, Langer TM, Álvarez-Salgado XA, Herndl GJ (2018). Dissolved organic carbon leaching from plastics stimulates microbial activity in the ocean. Nat. Commun..

[18] Albertsson A-C, Karlsson S (1988). The three stages in degradation of polymers: polyethylene as a model substance. J. Appl. Polym. Sci..

[19] Khaksar M-R, Ghazi-Khansari M (2009). Determination of migration monomer styrene from GPPS (general purpose polystyrene) and HIPS (high impact polystyrene) cups to hot drinks. Toxicol. Mech. Methods.

[20] Fayad NM, Sheikheldin SY, Al‐Malack MH, El‐Mubarak AH, Khaja N (1997). Migration of vinyl chloride monomer (VCM) and additives into PVC bottled drinking water. J. Environ. Sci. Heal.. Part A Environ. Sci. Eng. Toxicol..

[21] Akovali, G. Plastic materials: polyvinyl chloride (PVC). In *Toxicity of BuildingMaterials* 23–53, 10.1533/9780857096357.23 (Elsevier Inc, 2012).

[22] Kloukos D, Pandis N, Eliades T (2013). Bisphenol-A and residual monomer leaching from orthodontic adhesive resins and polycarbonate brackets: A systematic review. Am. J. Orthod. Dentofac. Orthop..

[23] Staniszewska M, Graca B, Nehring I (2016). The fate of bisphenol A, 4-tert-octylphenol and 4-nonylphenol leached from plastic debris into marine water – experimental studies on biodegradation and sorption on suspended particulate matter and nano-TiO2. Chemosphere.

[24] Moore JC (1964). Gel permeation chromatography. I. A new method for molecular weight distribution of high polymers. J. Polym. Sci. Part A Gen. Pap..

[25] Kesy K (2017). Fate and stability of polyamide-associated bacterial assemblages after their passage through the digestive tract of the blue mussel Mytilus edulis. Mar. Pollut. Bull..

[26] Harrison JP, Schratzberger M, Sapp M, Osborn AM (2014). Rapid bacterial colonization of low-density polyethylene microplastics in coastal sediment microcosms. BMC Microbiol..

[27] Tuma SN, Orson F, Fossella FV, Waidhofer W (1981). Seizures and dermatitis after exposure to caprolactam. Arch. Intern. Med..

[28] Sheldon T (1989). Chromosomal damage induced by caprolactam in human lymphocytes. Mutat. Res. Toxicol..

[29] Cruzan G (1998). Chronic Toxicity/Oncogenicity Study of Styrene in CD Rats by Inhalation Exposure for 104 Weeks. Toxicol. Sci..

[30] Vom Saal FS, Hughes C (2005). An extensive new literature concerning low-dose effects of bisphenol A shows the need for a new risk assessment. Environ. Health Perspect..

[31] Wright SL, Kelly FJ (2017). Plastic and Human Health: A Micro Issue?. Environ. Sci. Technol..

[32] Bruns A, Cypionka H, Overmann J (2002). Cyclic AMP and Acyl Homoserine Lactones Increase the Cultivation Efficiency of Heterotrophic Bacteria from the Central Baltic Sea. Appl. Environ. Microbiol..

[33] ASTM International. ASTM D6866-12: *Standard Test Methods for Determining the Biobased Content of Solid, Liquid, and Gaseous Samples Using Radiocarbon Analysis*. (ASTM International, 2012).

[34] International Organization of Standardization (ISO). *EN ISO 6427: Plastics - Determination of matter extractable by organic solvents (conventional methods)*. (ISO, 2013).

[35] Dickson, A. G., Sabine, C. L. & Christian, J. R. Guide to Best Practices for Ocean CO_2_ Measurements. *PICES Special Publ* 3, (North Pacific Marine Science Organization, 2007).

[36] R Core Team R: *a language and environment for statistical computing. R Foundation for Statistical Computing*. Vienna, Austria. URL. http://www.R-project.org. (2013).

[37] De Mendiburu F (2009). Agricolae: Statistical Procedures for Agricultural Research. R package version.

[38] Dinno, A. Dunn.test: Dunn’s Test of Multiple Comparisons Using Rank Sums. *R package version* 1.3.5. (2017).

