# Supplementary material for: Residual Monomer Content Affects the Interpretation of Plastic Degradation
Source: Sci Rep. 2019 Feb 14;9:2120. doi: 10.1038/s41598-019-38685-6 (PMC6375983; doi:10.1038/s41598-019-38685-6)
Supplement: Supplementary file 1 — Supplementary Information [file 41598_2019_38685_MOESM1_ESM.docx]

Supplementary material

**Residual Monomer Content Affects the Interpretation of Plastic Degradation**

Franziska Klaeger^1^ and Alexander S. Tagg^1^, Stefan Otto^1^, Matthias Bienmüller^2^, Ingo Sartorius^3^, Matthias Labrenz^1*^

**Artificial brackish water**

Artificial brackish water medium was prepared as followed:

*Stock solutions*

Table 1 Components and their amounts for the stock solutions with their final concentration.

| **Chemical** | **Applied weight [g]** | **Volume of  ultrapure water [ml]** | **Final concentration [mol/L]** |
| --- | --- | --- | --- |
| KBr | 9.997 | 100 | 0.84 |
| H_3_BO_3_ | 2.47 | 100 | 0.4 |
| SrCl x 6 H_2_O | 3.99 | 100 | 0.17 |
| NH_4_Cl | 2.14 | 100 | 0.4 |
| NaF | 0.294 | 100 | 0.07 |

All stock solutions were autoclaved and stored at 15 °C to the point of use.

*10* × *ABW medium*

Table 2 Components and their amounts for the 10 × ABW medium with their final concentration.

| **Chemical** | **Applied weight or volume** | **Final concentration  [mmol/L]** |
| --- | --- | --- |
| NaCl | 55.5 g | 950 |
| MgCl_2_ x 6 H_2_O | 22.9 g | 112.3 |
| CaCl_2_ x 2 H_2_O | 3.4 g | 23.13 |
| KCl | 1.5 g | 20.12 |
| Na_2_SO_4_ | 9.1 g | 64.1 |
| KBr stock solution | 2.29 ml | 1.92 |
| H_3_BO_3_ stock solution | 2.29 ml | 0.92 |
| SrCl stock solution | 2.29 ml | 0.11 |
| NH_4_Cl stock solution | 2.29 ml | 0.92 |
| NaF stock solution | 2.29 ml | 0.16 |
| Ultrapure water | 1000 ml ad |  |

The solution was diluted with ultrapure water (MilliQ, Merck KGaA *Darmstadt, Germany*) to 1 × ABW. Further, it was autoclaved and stored at 15°C to the point of use.

*Vitamin stock solution*

Vitamin B12 stock solution: 500µg of cyanocobalamin were dissolved in 1 ml of ultrapure water. The concentration was 0.369 mmol/L.

Table 3 Components and their amounts for the vitamin stock solution with their final concentration.

| **Chemical** | **Applied weight or volume** | **Final concentration  [mmol/L]** |
| --- | --- | --- |
| p-amino benzoic acid | 2.5 mg | 0.18 |
| Folic acid | 1 mg | 0.023 |
| Biotin | 1 mg | 0.041 |
| Nicotinic acid | 2.5 mg | 0.203 |
| Calcium pantothenate | 2.5 mg | 0.052 |
| Riboflavin | 2.5 mg | 0.066 |
| Thiamine HCl | 2.5 mg | 0.074 |
| Pyridoxine HCl  (Vitamin B6) | 5 mg | 0.243 |
| Cyanocobalamin  (Vitamin B12) stock solution | 0.1 ml | 0.00037 |
| Lipoic acid | 2.5 mg | 0.121 |
| Ultrapure water | 100 ml ad |  |

The solution was filtered through a 0.2 µm filter into a sterile falcon tube. It was stored at -20 °C in the dark. 2 ml of the vitamin stock solution was added to 1 L of 1 × ABW.

*Selenite and Tungstate stock solution*

Selenite stock solution: 6 mg Na_2_SeO_3_ were dissolved in 1 ml ultrapure water to a final concentration of 0.04 mol/L.

Tungstate stock solution: 8 mg Na_2_WO_4_ were dissolved in 1 ml ultrapure water to a final concentration of 0.03 mol/L.

**Table 4 Components and their amounts of the selenite tungstate stock solution with their final concentration.**

| **Chemical** | **Applied weight or volume** | **Final concentration [mmol/L]** |
| --- | --- | --- |
| NaOH | 40 mg | 10 |
| Selenite stock solution | 0.1 ml | 0.04 |
| Tungstate stock solution | 0.1 ml | 0.03 |
| Ultrapure water | 100 ml ad |  |

The solution was autoclaved and stored at 15 °C. 0.2 ml of it was added to 1 L of 1 × ABW.

*Trace elements SL10 stock solution*

Boric acid: 6 mg H_3_BO_3_ were dissolved in 1 ml ultrapure water to a final concentration of 0.097 mol/l.

Copper (II) chloride stock solution: 2 mg CuCl2 were dissolved in 1 ml ultrapure water to a final concentration of 0.015 mol/L.

**Table 5 Components and their amounts of the trace elements SL 10 stock solution with their final concentration.**

| **Chemical** | **Applied weight or volume** | **Final concentration [mmol/L]** |
| --- | --- | --- |
| CoCl_2_ x 6 H_2_O | 19 mg | 0,799 |
| MnCl_2_ x 2 H_2_O | 10 mg | 0.618 |
| ZnCl_2_ | 7 mg | 0.514 |
| Na_2_MO_4_ | 3.6 mg | 0,175 |
| NiCl_2_ | 2.4 mg | 0.185 |
| H_3_BO_3_ stock solution | 0.1ml | 0.097 |
| CuCl_2_ stock solution | 0.1ml | 0.015 |
| Ultrapure water | 100 ml ad |  |

The solution was autoclaved and stored at 15 °C. 1 ml of it was added to 1 L of 1 × ABW.

*Potassium dihydrogen orthophosphate stock solution*

**Table 6 Components and their amounts of the potassium dihydrogen orthophosphate stock solution with the final concentration.**

| **Chemical** | **Applied weight [g]** | **Final concentration [mol/L]** |
| --- | --- | --- |
| KH_2_PO_4_ | 2.45 | 0.18 |
| Ultrapure water | 100 ml ad |  |

The solution was autoclaved and stored at 15 °C. 5 ml of the stock solution was added to 1 L of 1 × ABW medium to have a final concentration of KH_2_PO_4_ of 0.0009 mol/L.

*Sodium bicarbonate stock solution*

**Table 7 Components and their amounts of the sodium bicarbonate solution with the final concentration.**

| **Chemical** | **Applied weight [g]** | **Final concentration [mol/L]** |
| --- | --- | --- |
| NaHCO_3_ | 1.3 | 0.62 |
| Ultrapure water | 25 ml ad |  |

The solution was autoclaved and stored at 15 °C. 2.5 ml of the stock solution was added to 1 L of 1 × ABW medium to have a final concentration of NaHCO_3_ of 0.0015 mol/L.

*Sodium carbonate stock solution*

**Table 8 Components and their amounts of the sodium carbonate solution with the final concentration.**

| **Chemical** | **Applied weight [g]** | **Final concentration [mol/L]** |
| --- | --- | --- |
| Na_2_CO_3_ | 0.082 | 0.0309 |
| Ultrapure water | 25 ml ad |  |

The solution was autoclaved and stored at 15 °C. 2.5 ml of the stock solution was added to 1 L of 1 × ABW medium to have a final concentration of Na_2_CO_3_ of 0.077 mmol/L ABW.

**Dissolved inorganic carbon**

**~1 % rM content**

Table 9 Labelling of the different size classes of the PA6.

| **Labelling** | **Size class** |
| --- | --- |
| PA6 A | < 125 µm |
| PA6 B | < 250 µm |
| PA6 C | < 500 µm |
| PA6 D | < 1500 µm |
| PA6 E | ~4000 µm |

Table 10 DIC values for samples with ~1 % rM content.

| **Sample type** | **DIC [µmol/kg]** |
| --- | --- |
| t0 start value | 827,08 |
| t0 start value | 827,61 |
| t0 start value | 829,65 |
| t34d PA6 A | 2409,53 |
| t34d PA6 A | 3180,71 |
| t34d PA6 A | 3176,92 |
| t34d PA6 B | 7417,39 |
| t34d PA6 B | 8318,58 |
| t34d PA6 B | 4494,05 |
| t34d PA6 C | 2244,48 |
| t34d PA6 C | 2338,95 |
| t34d PA6 C | 2932,72 |
| t34d PA6 D | 3073,38 |
| t34d PA6 D | 4326,51 |
| t34d PA6 D | 2422,89 |
| t34d PA6 E | 2651,9 |
| t34d PA6 E | 3461,13 |
| t34d PA6 E | 2610,32 |
| t34d C1 no added PA6 1 | 887,58 |
| t34d C1 no added PA6 2 | 816,23 |

Table 11 Oxygen values for 1 % rM content samples.

| **Sample type** | **O_2_ content [%]** |
| --- | --- |
| PA6 A | 90,4 |
| PA6 B | 88 |
| PA6 C | 93 |
| PA6 D | 19 |
| PA6 E | 79,4 |

Table 12 Comparison of Ultrapure water with samples with ultrapure water and PA6 1% rM.

| **Sample type** | **DIC [µmol/kg]** |
| --- | --- |
| Ultrapure water | 26,72 |
| PA6 A 1 | 69,29 |
| PA6 A 2 | 66,43 |

**~0.05 % and ~0.1 % rM content**

Table 13 DIC values for samples with ~0.05 % and ~0.1 % rM content after 19, 34 and 84 days.

| **Sample type** | **DIC [µmol/kg]** |
| --- | --- |
| t0 start value 1 | 784,36 |
| t0 start value 2 | 797,97 |
| t0 start value 3 | 802 |
| t19d PA6 ~0.1 % 1 | 1692,58 |
| t19d PA6 ~0.1 % 2 | 1813,68 |
| t19d PA6 ~0.1 % 3 | 1651,26 |
| t34d PA6 ~0.1 % 1 | 2033,16 |
| t34d PA6 ~0.1 % 2 | 1392,78 |
| t34d PA6 ~0.1 % 3 | 2002,17 |
| t84d PA6 ~0.1 % 1 | 2120,31 |
| t84d PA6 ~0.1 % 2 | 1901,21 |
| t84d PA6 ~0.1 % 3 | 2154,17 |
| t19d PA6 ~0.05 % 1 | 1131,07 |
| t19d PA6 ~0.05 % 2 | 1153,54 |
| t19d PA6 ~0.05 % 3 | 1130,02 |
| t34d PA6 ~0.05 % 1 | 944,6 |
| t34d PA6 ~0.05 % 2 | 1201,83 |
| t34d PA6 ~0.05 % 3 | 948,95 |
| t84d PA6 ~0.05 % 1 | 1628,71 |
| t84d PA6 ~0.05 % 2 | 1055,32 |
| t84d PA6 ~0.05 % 3 | 1755,43 |
| t34d C1 no added PA6 1 | 858,02 |
| t34d C1 no added PA6 2 | 877,68 |

Table 14 Oxygen content of 0.1 % and 0.05 % rM content samples.

| **Sample type** | **O_2_ content [%]** |
| --- | --- |
| t19d PA6 ~0.1 % 1 | 78,5 |
| t19d PA6 ~0.1 % 2 | 87,95 |
| t19d PA6 ~0.05 % 1 | 94,2 |
| t19d PA6 ~0.05 % 2 | 100,85 |
| t34d PA6 ~0.1 % 1 | 91 |
| t34d PA6 ~0.1 % 2 | 75,7 |
| t34d PA6 ~0.05 % 1 | 90,45 |
| t34d PA6 ~0.05 % 2 | 100,8 |
| t84d PA6 ~0.1 % 1 | 93,4 |
| t84d PA6 ~0.1 % 2 | 95,5 |
| t84d PA6 ~0.05 % 1 | 77,6 |
